# Prophylactic pegfilgrastim to prevent febrile neutropenia among patients receiving biweekly (Q2W) chemotherapy regimens: a systematic review of efficacy, effectiveness and safety

**DOI:** 10.1186/s12885-021-08258-w

**Published:** 2021-05-27

**Authors:** Reshma Mahtani, Jeffrey Crawford, Sinéad M. Flannery, Tatiana Lawrence, Jennifer Schenfeld, Prasad L. Gawade

**Affiliations:** 1 Sylvester Cancer Center, University of Miami, Deerfield Beach, FL USA; 2 Duke Cancer Institute, Durham, NC USA; 3 Oxford PharmaGenesis Ltd, London, UK; 4 US Medical, Amgen, Thousand Oaks, CA USA; 5 Center for Observational Research, Amgen, Thousand Oaks, CA USA

**Keywords:** Pegfilgrastim, Febrile neutropenia, Biweekly, Q2W, G-CSF

## Abstract

**Background:**

Pegfilgrastim, a long-acting granulocyte colony-stimulating factor (G-CSF), is commonly used to prevent febrile neutropenia (FN), a potentially life-threatening complication, following myelosuppressive chemotherapy. The FDA label for pegfilgrastim specifies that it should not be administered 14 days before or within 24 h of administration of myelosuppressive chemotherapy, precluding the use of pegfilgrastim in biweekly (Q2W) regimens. The National Comprehensive Cancer Network and the European Organisation for Research and Treatment of Cancer guidelines support the use of prophylactic pegfilgrastim in patients receiving Q2W regimens. The objective of this study was to systematically review evidence from randomized clinical trials (RCTs) and observational studies that describe the effectiveness and safety of prophylactic pegfilgrastim in preventing FN among patients receiving Q2W regimens.

**Methods:**

An Ovid MEDLINE, Embase, and Cochrane Library literature search was conducted to evaluate the evidence regarding efficacy, effectiveness, and safety of prophylactic pegfilgrastim versus no prophylactic pegfilgrastim or prophylaxis with other G-CSF in patients who were receiving Q2W chemotherapy regimens with high (> 20%) or intermediate (10–20%) risk of FN for a non-myeloid malignancy. Studies that addressed absolute or relative risk of FN, grade 1–4 neutropenia, all-cause or any hospitalization, dose delays or dose reductions, adverse events, or mortality were included. Studies where the comparator was a Q3W chemotherapy regimen with primary prophylactic pegfilgrastim were also included.

**Results:**

The initial literature search identified 2258 publications. Thirteen publications met the eligibility criteria, including eight retrospective, one prospective, one phase 1 dose escalation study, and three RCTs. In nine of the 13 studies reporting incidence of FN, and in seven of the nine studies reporting incidence of neutropenia, administration of prophylactic pegfilgrastim in patients receiving Q2W regimens resulted in decreased or comparable rates of FN or neutropenia compared with patients receiving filgrastim, no G-CSF, lipefilgrastim or pegfilgrastim in Q3W regimens. In six of the nine studies reporting safety data, lower or comparable safety profiles were observed between pegfilgrastim and comparators.

**Conclusions:**

In a variety of non-myeloid malignancies, administration of prophylactic pegfilgrastim was efficacious in reducing the risk of FN in patients receiving high- or intermediate-risk Q2W regimens, with an acceptable safety profile.

**Trial registration:**

**PROSPERO registration no**: CRD42019155572.

**Supplementary Information:**

The online version contains supplementary material available at 10.1186/s12885-021-08258-w.

## Background

Febrile neutropenia (FN) is a potentially life-threatening complication that can occur after myelosuppressive chemotherapy, and is associated with a reduction in treatment efficacy because of dose delays and dose reductions [1–4]. Granulocyte colony-stimulating factors (G-CSF) are commonly used to prevent FN [5]. Treatment guidelines recommend the use of G-CSF as primary prophylaxis during chemotherapy if the risk of FN is greater than 20% [5–8]. Primary prophylaxis involves using G-CSF in the first and subsequent sessions of chemotherapy [9].

Pegfilgrastim is a long-acting G-CSF, indicated to decrease the incidence of infection manifested by FN and reduce the duration of neutropenia [10, 11]. It is administered once per cycle and is the most commonly used G-CSF in the USA [10]. The FDA label for pegfilgrastim specifies that it should not be administered 14 days before or within 24 h of administration of myelosuppressive chemotherapy [11]. This restriction was placed because of a theoretical potential to increase toxicity of chemotherapy to myeloid progenitor cells after growth factor stimulation [12]. Two studies have previously reported an increase in the incidence of grade 4 neutropenia in patients receiving five consecutive days of overlapping chemotherapy with filgrastim [13, 14]. Consequently, the restriction precludes the prophylactic use of pegfilgrastim with a biweekly (Q2W) chemotherapy regimen in the US. In contrast to the FDA label, the EMA label states that pegfilgrastim is recommended for each chemotherapy cycle, given at least 24 h after cytotoxic chemotherapy [15].

The National Comprehensive Cancer Network (NCCN) guidelines recommend at least 12 days between a dose of pegfilgrastim and the next cycle of chemotherapy, supporting the use of prophylactic pegfilgrastim in patients receiving a Q2W regimen [5]. This is consistent with the guidelines of the European Organisation for Research and Treatment of Cancer (EORTC), which state that pegfilgrastim can be administered with chemotherapy in patients receiving treatment at 14-day intervals [6]. There is, however, a lack of systematic appraisal of the current evidence base from randomized controlled trials (RCTs) and observational studies that summarizes the efficacy, effectiveness, and safety of prophylactic pegfilgrastim to prevent FN among patients treated with a Q2W chemotherapy regimen. Use of prophylactic pegfilgrastim in patients receiving a Q2W regimen can allow 14 days for hematologic recovery between treatment administrations which may improve efficacy without affecting safety, and has the potential to reduce the risk of FN and potential costly hospitalization for FN; therefore improving a patient’s quality of life [6]. The objective of this review was to examine whether prophylactic pegfilgrastim treatment is efficacious and has an acceptable and comparable safety profile compared with prophylaxis with other G-CSFs (both short-acting and long-acting), in reducing the risk of FN among patients treated with a Q2W chemotherapy regimen who have a high or intermediate risk of FN.

## Methods

### Search strategy

The systematic literature review protocol was pre-registered in PROSPERO: CRD42019155572 [16]. An Ovid MEDLINE, Embase, and Cochrane Library literature search was performed using the search terms included in Supplemental Table S1–3 (Additional file 1). The literature search included studies from January 1, 2002 to June 30, 2019. A congress abstract literature search was performed for abstracts published in key international congresses (Supplemental Table S4 [Additional file 1]) held from June 30, 2016 to June 30, 2019. The congress abstract literature search was limited to the preceding 3 years because abstracts before this time frame were likely to be superseded by full-length texts previously captured in the Ovid MEDLINE and Embase literature search.

### Population, intervention, comparison, and outcomes (PICOS) criteria

The population, intervention, comparison, and outcomes (PICOS) criteria for the inclusion and exclusion criteria of studies were as follows. The population was participants diagnosed with a non-myeloid malignancy and treated with a Q2W chemotherapy regimen; studies in which the comparator was a Q3W chemotherapy regimen plus primary prophylactic pegfilgrastim were also included. Interventions were Q2W myelosuppressive chemotherapy regimens associated with a high (> 20%) or intermediate (10–20%) risk of FN plus prophylactic pegfilgrastim. Comparisons included prophylactic pegfilgrastim for the Q3W studies, primary prophylaxis with a G-CSF other than pegfilgrastim, no prophylactic G-CSF or placebo. In RCTs, FN has been defined as an absolute neutrophil count of less than 0.5 × 10^9^/L, or of less than 1.0 × 10^9^/L that is predicted to fall to less than 0.5 × 10^9^/L within 48 h, with fever or clinical signs of sepsis. For observational studies, FN is commonly defined as an in-patient stay with a diagnosis claim for neutropenia, fever, or infection. However, we did not exclude studies if the definition of FN was a variant of the commonly used definitions of FN presented above. Additional outcomes were grade 1–4 neutropenia, all-cause or any hospitalization, dose delays or dose reductions, dose delays or dose reductions as a result of neutropenia, adverse events (AEs), and mortality.

Exclusion criteria included single-arm trials, observational studies with no control or comparison group, studies of patients with myeloid malignancy, animal studies, abstracts and other publications superseded by more recent publications, editorials, and letters to editors. Observational studies with fewer than 30 patients in the Q2W arm with primary prophylactic pegfilgrastim were excluded.

### Analysis

Two reviewers independently screened the titles and abstracts of all publications retrieved to determine eligibility according to the PICOS criteria, and any conflicts were resolved by a third reviewer. Of the studies identified for inclusion, the full publications were retrieved and reviewed by two authors to confirm eligibility. A third review of the full text of selected studies was performed to assess eligibility and risk of bias.

The risk of bias tool recommended by the Cochrane Collaboration was used for RCTs [17]. This tool addresses six domains: selection bias, performance bias, detection bias, attrition bias, reporting bias, and other biases. Within each domain, assessments are made for one or more items, which may cover different aspects of the domain or different outcomes. Each domain was graded as ‘low risk’, ‘high risk’, or ‘unclear risk’ of bias [17]. For the remaining studies, a quality assessment tool designed to assess bias in non-randomized observational studies, the Cochrane ROBINS-I tool, was used [18]. The ROBINS-I tool consists of seven domains which include bias due to: confounding, selection of participants into the study, measurement of interventions, deviations from intended interventions, missing outcome data, measurement of the outcome, and selection of the reported results. Each domain was graded as ‘low’, ‘moderate’, ‘serious’, or ‘critical’ risk of bias [18].

Given the heterogeneity of the studies in terms of tumor types, FN risk associated with chemotherapy regimens, and comorbidity profile of patient populations, meta-analyses were not conducted.

## Results

**Fig. 1:**
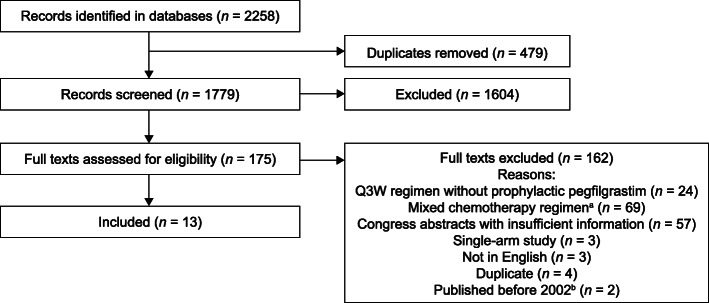
PRISMA diagram. ^a^Mixed chemotherapy regimens which included both Q2W and Q3W studies without prophylactic pegfilgrastim (or patients received both Q2W and Q3W regimens) were excluded. ^b^The literature search included all relevant studies from January 1, 2002 through June 30, 2019. Any study before 2002 was then excluded. *PRISMA* Preferred Reporting Items for Systematic Reviews and Meta-Analyses, *Q2W* biweekly, *Q3W* every 3 weeks

The initial literature search identified 2258 publications from Ovid MEDLINE, Embase, the Cochrane Library, and congress searches (Fig. 1). Of these, 479 were found to be duplicates and were eliminated. After duplicates were removed, the titles and abstracts of 1779 records were screened against the PICOS criteria; 1604 records were subsequently removed.

Full-text review was performed on the remaining 175 publications, of which 162 were removed for the following reasons: Q3W regimen without prophylactic pegfilgrastim (*n* = 24); mixed chemotherapy regimen, that is, a chemotherapy regimen that included both Q2W and Q3W administration without prophylactic pegfilgrastim (*n* = 69); congress abstracts with insufficient information (*n* = 57); single-arm studies (*n* = 3); not in the English language (*n* = 3); duplicate studies (*n* = 4), or published before 2002 (*n* = 2) (Fig. 1).

In total, 13 studies met the eligibility criteria and were included as part of the systematic literature review (Table 1) [19–31]. There were eight retrospective cohort studies [19, 21, 25–29, 31], one prospective cohort study [24], one phase 1 dose escalation study [22], and three RCTs [20, 23, 30]. Tumor types included breast cancer, lymphoma, colon cancer, rectal cancer, gastric cancer, pancreatic cancer, esophageal cancer, and small bowel cancer. The most commonly used chemotherapy regimens reported in the eligible studies were cyclophosphamide, doxorubicin, vincristine, and prednisone (CHOP); rituximab and CHOP (R-CHOP); 5-fluorouracil, leucovorin, and oxaliplatin (FOLFOX); 5-fluorouracil, leucovorin, and irinotecan (FOLFIRI); 5-fluorouracil, leucovorin, oxaliplatin, and irinotecan (FOIL); gemcitabine with increasing doses of docetaxel; doxorubicin and cyclophosphamide (AC); and hyper-fractionated cyclophosphamide, vincristine, doxorubicin, and dexamethasone (hyper-CVAD) (Table 1).

**Table 1:** Literature search results – included studies

Study	Patients enrolled	Study design	Data source	Year	Location	Comparator	Cancer type	Chemotherapy regimen	Study objectives
Balducci [19]	*N* = 199	Retrospective analysis	Five Amgen-sponsored pegfilgrastim clinical trials: 1. NCT00117897 2. Amgen study number 20010203 3. Amgen study number 990118 4. NCT002771605. NCT00115193	n/a (combined analyses)	n/a (combined analyses)	Q3W regimen	NHL	CHOP or R-CHOP	Examine the impact of pegfilgrastim as primary prophylaxis on maintaining RDI in patients with NHL treated with CHOP-based chemotherapy Evaluate the incidence of chemotherapy dose delays, dose reductions, treatment discontinuation, and AEs leading to dose alteration (i.e., dose reduction or dose delay) or treatment discontinuation
Bozzoli [20]	*N* = 51	Prospective, randomized study	n/a	Jan 2006–Sep 2011	Europe (Italy)	Filgrastim	DLBCL	R-CHOP-14	The dose intensity of chemotherapy during the first four R-CHOP-14 cycles Secondary endpoints: incidence of adverse reactions, FN, and hospitalization, and the response to chemotherapy
Donkor [21]	*N* = 51	Single-institution, retrospective cohort	Cancer center of a 900-bed academic medical center	Jun 2013–Dec 2015	n/a	CSF or none	Colon, rectal, appendix, gastric, pancreatic, esophageal, small bowel	FOLFOX ± MAB, FOLFIRI ± MAB, FOLFIRINOX, or other 5-FU infusion-containing regimens ± MAB	Assess the number of chemotherapy cycles with neutropenia, FN, and/or hospitalizations in cycles in which pegfilgrastim was administered < 14 days from the next chemotherapy dose
Dragnev [22]	*N* = 35	Single-institution, open-label, dose-escalation, phase 1 trial	n/a	Apr 2000–Aug 2004	USA	Filgrastim	Solid tumors with a preponderance of GI cancer and pancreaticobiliary tumors	Gemcitabine 3000 mg/m^2^ and increasing doses of docetaxel (55 mg/m^2^ in 10 mg/m^2^ increments) every 14 days	Establish the maximum tolerated dose of docetaxel given with gemcitabine 3000 mg/m^2^ on a Q2W schedule with growth factor support Determine the feasibility of using filgrastim or pegfilgrastim to increase the dose intensity of Q2W docetaxel and gemcitabine Secondary endpoints: safety of administration of pegfilgrastim with a Q2W chemotherapy regimen; exploratory comparison between the efficacy and safety of filgrastim and pegfilgrastim with docetaxel and gemcitabine every 14 days
Hecht [23]	*N* = 252	1:1 randomized, double-blind, placebo-controlled, phase 2 trial	n/a	Feb 2003–Mar 2008	USA	Placebo	CRC	Patients received one of the following three Q2W chemotherapy regimens: FOLFOX-4: oxaliplatin 85 mg/m^2^ on day 1, LV 200 mg/m^2^ IV on days 1 and 2, 5-FU 400 mg/m^2^ bolus, then 600 mg/m^2^ administered over 22 h on both days 1 and 2 FOLFIRI: irinotecan 180 mg/m^2^ on day 1, LV 200 mg/m^2^ IV on days 1 and 2, 5-FU 400 mg/m^2^ bolus, then 600 mg/m^2^ administered over 22 h on both days 1 and 2 FOIL: irinotecan 175 mg/m^2^ IV on day 1, oxaliplatin 100 mg/m^2^ IV on day 1, LV 200 mg/m^2^ IV on day 1, 5-FU 3.0 g/m^2^ IV administered over 48 h starting on day 1	Incidence of grade 3/4 neutropenia (ANC < 1.0 × 10 ^× 9^/L) during the first four cycles of chemotherapy Secondary endpoints: incidence of grade 3/4 FN, neutropenia-related hospitalization rate and related antibiotic use, incidence of chemotherapy dose delays and/or dose reductions because of a neutropenic event, ORR, and incidence of AEs
Hendler [24]	*N* = 231	Prospective, non-randomized trial	CALGB study	Jun 2003–Jan 2009	n/a	Filgrastim	Breast cancer	Four cycles of doxorubicin (60 mg/m^2^) and cyclophosphamide (600 mg/m^2^; AC) once Q2W for four cycles, followed by weekly paclitaxel (80 mg/m^2^) for 12 weeks	Evaluate the four different schedules of growth factor support administration with regard to the occurrence of FN, hospitalization events, treatment delays, and other hematological toxicities
Kourlaba [25]	*N* = 1058	Retrospective cohort	Two randomized trials (HE10/00 and HE10/05) and an observational study (HE10/08)	n/a	Data analyzed in Greece	Filgrastim	Breast cancer	For HE10/00: patients randomized to receive either epirubicin (E, 110 mg/m^2^) Q2W for three cycles, followed by three cycles of paclitaxel (T, 250 mg/m^2^) Q2W, followed by three cycles of CMF (cyclophosphamide 840 mg/m^2^, methotrexate 57 mg/m^2^ and 5-FU 840 mg/m^2^) Q2W (E-T-CMF) for group A or E (83/m^2^) in combination with T (187/m^2^) Q3W for four cycles, followed by CMF as above Q2W for three cycles (ET-CMF) for group B For HE10/05: patients were randomized to receive E-T-CMF as in group A of protocol HE10/00 for group A or E (110 mg/m^2^) Q2W for three cycles, followed by three cycles of CMF (cyclophosphamide 840 mg/m^2^, methotrexate 57 mg/m^2^ and 5-FU 840 mg/m^2^) Q2W followed 3 weeks later by nine weekly cycles of docetaxel 35 mg/m^2^ (E-CMF-DOC) for group B or E-CMF (as previously described in group B) followed 3 weeks later by nine weekly cycles of paclitaxel 80 mg/m^2^ for group For HE10/08: 110 mg/m^2^ Q2W for three cycles, followed by three cycles of paclitaxel (T, 200 mg/m^2^) Q2W followed by three cycles of CMF (cyclophosphamide 600 mg/m^2^, methotrexate 45 mg/m^2^ and 5-FU 600 mg/m^2^) Q2W (E-T-CMF)	Primary endpoints were FN, severe (grade 3, 4) neutropenia, dose reduction (> 10% reduction of the dose planned), and treatment delay (dose given > 2 days later)
Kurbacher [26]	*N* = 53	Retrospective analysis	Not reported	n/a	n/a	Lipegfilgrastim	Breast or gynecologic cancer	1–4 ddCtx	Use of the long-acting G-CSFs as FN primary prophylaxis in the clinical routine
Lane [27]	*N* = 81	Multicenter, retrospective analysis	Audit from databases of Australian hospitals^a^	Jan 1999–Jul 2005	Australia	Filgrastim	ALL or NHL	Hyper-CVAD chemotherapy	Duration of grade 4 neutropenia (ANC < 500/μL)
Lugtenburg [28]	*N* = 1113	Multicenter, retrospective/prospective, observational study	IMPACT NHL study (NCT00903812)	Patients had to have started chemotherapy after Jan 1, 2005	Europe and Australia	CHOP every 21 days	DLBCL	Rituximab (R-CHOP) every 14 days or every 21 days	Evaluate the impact of age group (younger, < 65 years old; older, ≥ 65 years old) on the assessment of FN risk, G-CSF use patterns, incidence of FN, and chemotherapy delivery in patients with DLBCL receiving an R-CHOP regimen Outcome measures: the proportion of patients in whom investigators assessed risk of FN at ≥20% and who received primary prophylaxis with G-CSF, as well as type of G-CSF, rate of FN, chemotherapy relative dose intensity ≥90%, chemotherapy dose delays, and chemotherapy dose reductions
Ng [29]	*N* = 132	Single-center, observational, retrospective cohort	National Cancer Centre Singapore, patients identified through the Singapore Lymphoma Registry	Jan 2007–May 2009	Singapore	CHOP every 21 days	NHL	CHOP every 21 days (standard dose regimen, designated as CHOP-21) or every 14 days (dose-dense regimen designated as CHOP-14)	Identify clinical characteristics of patients on CHOP-based chemotherapy that would predispose them to develop breakthrough FN and provide descriptive data on the incidence of breakthrough FN among patients with lymphoma
Pinter [30]	*N* = 845	Phase 3, double-blind randomized trial	Pegfilgrastim and Anti-VEGF Evaluation Study (PAVES)	Nov 2009–Jan 2012	North America and rest of world^b^	Placebo	Locally advanced or metastatic adenocarcinoma of the colon or rectum	Physicians predetermined the chemotherapy regimen that was included with bevacizumab (5 mg/kg intravenous infusion on day 1 of each 14-day cycle), either FOLFOX (FOLFOX4, FOLFOX6 or modified mFOLFOX6) or FOLFIRI (FOLFIRI [Douillard] or FOLFIRI)	The incidence of grade 3/4 FN during the first four cycles Other endpoints: the incidence of grade 4 FN, grade 3/4 neutropenia, grade 4 neutropenia, CSF use, RDI, dose delays, and dose reductions during the four treatment cycles and ORR, OS, and PFS during the LTFU period
Skarlos [31]	*N* = 214	Retrospective (non-randomized), matched case-control study	Two randomized trials (HE10/00 and HE10/05) from the Hellenic Cooperative Oncology Group	Not reported	Data analyzed in Greece	Filgrastim on day 2–10 following chemotherapy	Histologically confirmed epithelial breast cancer	ET-CMF: Epirubicin (E) every 2 weeks for 3 cycles followed by 3 cycles of Paclitaxel (T) every 2 weeks, followed by 3 cycles of CMF (cyclophosphamide, methotrexate, and 5-Fluorouracil) every 2 weeks	Proportion of patients with early breast cancer who developed FN among those who received filgrastim or pegfilgrastim support during adjuvant treatment with dose-dense sequential therapy Secondary endpoints: the incidence of severe (grade 3, 4) neutropenia and the ability to receive chemotherapy as planned (≤ 90% of the dose planned and no dose given > 2 days later)

^a^The following hospitals were included: The Princess Alexandra Hospital Brisbane, Sir Charles Gairdner Hospital Perth and the Peter MacCallum Cancer Centre, Melbourne, Australia

^b^North America included both Canada and the USA. The rest of the world includes Austria, Belgium, Czech Republic, France, Hungary, Ireland, Italy, Latvia, Mexico, Poland, Romania, Russian Federation, Slovakia and Ukraine

*5-FU* 5-fluorouracil, *AE* adverse event, *ALL* acute lymphoblastic leukemia, *ANC* absolute neutrophil count, *CALGB* Cancer and Leukemia Group B, *CHOP* cyclophosphamide, doxorubicin, vincristine, and prednisone, *CRC* colorectal cancer, *CSF* colony-stimulating factor, *DLBCL* diffuse large B-cell lymphoma, *FN* febrile neutropenia, *FOIL* 5-FU, LV, oxaliplatin, and irinotecan, *FOLFIRI* 5-FU, LV, and irinotecan, *FOLFIRINOX* 5-FU, LV, irinotecan, and oxaliplatin, *FOLFOX* 5-FU, LV, and oxaliplatin, *G-CSF* granulocyte colony-stimulating factor, *GI* gastrointestinal, *IV* intravenously, *LTFU* loss to follow-up, *LV* leucovorin, *MAB* monoclonal antibody, *n/a* not applicable, *NHL* non-Hodgkin’s lymphoma, *ORR* overall response rate, *OS* overall survival, *PFS* progression-free survival, *PP* prophylactic pegfilgrastim, *Q2W* biweekly, *Q3W* every 3 weeks, *R-CHOP* rituximab, cyclophosphamide, doxorubicin, vincristine, and prednisone, *RDI* relative dose intensity, *VEGF* vascular endothelial growth factor

Six studies evaluated filgrastim versus pegfilgrastim administration [20, 22, 24, 25, 27, 31], two studies evaluated placebo versus pegfilgrastim [23, 30], one study evaluated filgrastim versus pegfilgrastim or no prophylactic G-CSF versus pegfilgrastim [21], and one study evaluated lipegfilgrastim versus pegfilgrastim [26]. Three studies evaluated outcomes following prophylactic pegfilgrastim among patients receiving a Q3W regimen with those receiving Q2W regimen [19, 28, 29].

### Studies reporting febrile neutropenia

All 13 eligible studies (three RCTs, 10 non-randomized) reported the incidence of FN (Table 2) [19–31]. The definitions and timing of assessment of FN are described in Supplemental Table S5 (Additional file 1). In the two RCTs that compared placebo versus pegfilgrastim in patients with colorectal cancer (Hecht et al. and Pinter et al.) receiving FOLFOX, FOLFIRI or FOIL, both studies showed a significantly lower incidence of FN with pegfilgrastim compared with placebo [23, 30]. In the RCT that compared filgrastim versus pegfilgrastim in patients with DLBCL receiving R-CHOP-14 (Bozzoli et al.), a numerically lower incidence of FN was observed with pegfilgrastim compared with filgrastim, however the differences observed in the incidence of FN were not statistically significant (*p* < .05) [20].

**Table 2:** Incidence of febrile neutropenia

Study	Febrile neutropenia								
Balducci [19]		Combined CHOP and R-CHOP Q2W				Combined CHOP and R-CHOP Q3W			
		< 65 years	65–75 years	> 75 years	Overall	< 65 years	65–75 years	> 75 years	Overall
	Any grade	2 (6.3)	1 (3.3)	–	3 (4.8)	0 (0)	2 (2.6)	1 (3.1)	3 (2.2)
	Grade ≥ 3	2 (6.3)	1 (3.3)	–	3 (4.8)	0 (0)	1 (1.3)	1 (3.1)	2 (1.5)
Bozzoli [20]		Total (*n* = 51)		Pegfilgrastim (*n* = 27)		Filgrastim (*n* = 24)			*p* value
	FN per patient, *n* (%)	9/51 (18)		4/27 (15)		5/24 (21)			0.7
	FN per cycle, *n* (%)	13/201 (6.5)		6/105 (5.7)		7/96 (7.2)			0.8
Donkor [21]		Pegfilgrastim < 14 days group (*n* = 126)		Pegfilgrastim > 14 days group (*n* = 25)		Filgrastim group (*n* = 90)		No CSF group (*n* = 295)	
	FN per cycle	0 (0)		0 (0)		0 (0)		2 (0.7)	
Dragnev [22]		Filgrastim (*n* = 25)		Pegfilgrastim (*n* = 10)		*p* value			
	FN, *n*	0		0		*p* = 1.0			
Hecht [23]		Placebo		Pegfilgrastim		OR (95% CI)			
	Grade 3/4 FN, %	8		2		0.27 (0.07–1.00); *p* = 0.04			
Hendler^a^ [24]		Overall patients, *n* (%)		Group A	Group B	Group C		Group D	
	Total treated, *n* (%)	231 (100)		84 (36.3)	26 (11.3)	64 (27.7)		57 (24.7) 6 (10.5)	
	FN	13 (5.6)		3 (3.6)	3 (11.5)	1 (1.5)			
Kourlaba [25]		Filgrastim (95% CI)		Pegfilgrastim (95% CI)		*p* value			
	FN, % (95% CI)	3.4 (2.0–5.3)		4.3 (2.8–6.4)		*p* = 0.5			
Kurbacher [26]		Pegfilgrastim (*n* = 27)		Lipegfilgrastim (*n* = 26)					
	FN, %	2.2		0					
Lane [27]		Pegfilgrastim		G-CSF		*p* value			
	FN, %	29.0		37.9		*p* = 0.16			
Lugtenburg [28]		R-CHOP-14		R-CHOP-21					
		< 65 years (*n* = 241)	≥ 65 years (*n* = 168)	< 65 years (*n* = 343)		≥ 65 years (*n* = 363)			
	FN (any cycle), %	17	23	14		24			
	PP per intervention, %	54	58	17		32			
	Patients treated with pegfilgrastim with FN, %	9	13	2		8			
Ng [29]		CHOP-14 (*n* = 2)		CHOP-21 (*n* = 16)					
	Incidence of breakthrough FN, %	3.3		22.2					
Pinter [30]		Pegfilgrastim, *n* (%)	Placebo, *n* (%)	Difference	OR		*p* value		
	Grade 3/4 FN	10 (2.4)	24 (5.7)	−3.3%(−6.6, −0.0)	0.41 (0.19–0.86)		*p =* 0.014		
	Grade 4 FN	10 (2.4)	15 (3.5)	−1.2%(−4.0, 1.7)	0.66 (0.29–1.49)		*p =* 0.312		
	Grade 3/4 FN in FOLFOX-treated patients	2 (1.0)	13 (6.3)	n/a	0.15 (0.03–0.65)		n/a		
	Grade 3/4 FN in FOLFIRI-treated patients	8 (3.7)	11 (5.1)	n/a	0.72 (0.28–1.83)		n/a		
	Grade 3/4 FN in low-dose patients	3 (1.9)	7 (4)	n/a	0.46 (0.12–1.8)		n/a		
	Grade 3/4 FN in high-dose patients	7 (2.7)	17 (6.8)	n/a	0.37 (0.15–0.92)		n/a		
Skarlos [31]		Filgrastim		Pegfilgrastim		*p* value			
	FN, *n* (%)	1 (1)		14 (13)		*p* = 0.001			

^a^Group A: G-CSF 300 μg consecutive administrations during days 3–10; group B: G-CSF 300 μg consecutive administrations during days 3–7; group C: G-CSF administrations every other day for days 5, 7, 9, and 11; and group D: one administration of pegfilgrastim 6 mg on day 2

*CHOP* cyclophosphamide, doxorubicin, vincristine, and prednisone, *CI* confidence interval, *CSF* colony-stimulating factor, *FN* febrile neutropenia, *FOLFIRI* 5-fluorouracil, leucovorin, and irinotecan, *FOLFOX* 5-fluorouracil, leucovorin, and oxaliplatin, *G-CSF* granulocyte colony-stimulating factor, *n/a* not applicable, *OR* odds ratio, *PP* prophylactic pegfilgrastim, *Q2W* biweekly, *Q3W* every 3 weeks, *R-CHOP* rituximab, cyclophosphamide, doxorubicin, vincristine, and prednisone

Among the 10 non-randomized studies, five studies evaluated filgrastim versus pegfilgrastim [22, 24, 25, 27, 31], one study (Donkor et al.) evaluated filgrastim or no prophylactic G-CSF versus pegfilgrastim [21], one study (Kurbacher et al.) evaluated lipegfilgrastim versus pegfilgrastim [26], and three studies (Balducci et al., Lugtenburg et al. and Ng et al.) evaluated patients receiving a Q3W regimen and prophylactic pegfilgrastim versus a Q2W regimen and prophylactic pegfilgrastim [19, 28, 29]. Four out of five studies showed a lower, comparable, or not statistically significant different incidence of FN with pegfilgrastim compared with filgrastim [22, 24, 25, 27]. In Skarlos et al., a post-hoc non-randomized subgroup analysis evaluating filgrastim versus pegfilgrastim, a significantly higher incidence of FN was observed in patients treated with pegfilgrastim compared with those receiving filgrastim (13% vs. 1% for pegfilgrastim vs. filgrastim, respectively) [31]. In Donkor et al. which evaluated pegfilgrastim versus filgrastim versus no prophylactic G-CSF, a numerically lower or comparable incidence of FN was observed with pegfilgrastim compared with filgrastim or no prophylactic G-CSF (0.0% vs. 0.0% vs. 0.7% for pegfilgrastim vs. filgrastim vs. no prophylactic G-CSF, respectively); statistical significance was not assessed in this study [21].

Two out of three studies showed a non-statistically significant lower or comparable incidence of FN with a Q2W regimen and prophylactic pegfilgrastim compared with a Q3W regimen and prophylactic pegfilgrastim [19, 29]. In Kurbacher et al., a study evaluating lipegfilgrastim versus pegfilgrastim, a numerically higher incidence of FN was observed in patients treated with pegfilgrastim compared with those receiving lipegfilgrastim (2.2% vs. 0.0% for pegfilgrastim vs. lipegfilgrastim); of note, statistical significance was not measured for this outcome in this study [26].

### Studies reporting grade 1*–*4 neutropenia

Nine studies (two RCTs and seven retrospective cohort studies) reported the incidence of grade 1–4 neutropenia (Table 3) [19, 21–23]. In both RCTs, the incidence of neutropenia in pegfilgrastim arm was significantly lower than placebo arm [23, 30]. In one retrospective study (Kourlaba et al.), the incidence of neutropenia in pegfilgrastim arm was significantly lower than the filgrastim arm (*p* <  0.001) [25]. Among the six remaining retrospective cohort studies, three studies reported a lower incidence of grade 1–4 neutropenia following pegfilgrastim, when compared with filgrastim [21, 22], no G-CSF [21], or pegfilgrastim in Q3W regimen [19]; in two of these studies where a lower incidence was observed, statistical significance was not assessed [19, 21] In the other three studies, one study reported comparable but not statistically significant incidences of neutropenia following pegfilgrastim versus filgrastim (*p* = 0.55) [27], and two studies reported an increased risk of neutropenia following pegfilgrastim versus filgrastim (*p* = 0.36) [31] and lipefilgrastim (statistical significance was not assessed) [26].

**Table 3:** Incidence of grade 1–4 neutropenia

Study	Neutropenia								
Balducci [19]	Neutropenia	Combined CHOP and R-CHOP Q2W				Combined CHOP and R-CHOP Q3W			
		< 65 years	65–75 years	> 75 years	Overall	< 65 years	65–75 years	> 75 years	Overall
	Any grade, *n* (%)	0 (0)	1 (3.3)	–	1 (1.6)	0 (0)	5 (6.4)	1 (3.1)	6 (4.4)
	Grade ≥ 3, *n* (%)	0 (0)	0 (0)	–	0 (0)	0 (0)	2 (2.6)	1 (3.1)	3 (2.2)
Donkor [21]		Pegfilgrastim < 14 days group (*n* = 126)		Pegfilgrastim > 14 days group (*n* = 25)		Filgrastim group (*n* = 90)		No CSF group (*n* = 295)	
	Incidence of neutropenia, *n* (%)	0 (0)		1 (4)		24 (26.7)		25 (8.5)	
Dragnev [22]		Filgrastim (*n* = 25)		Pegfilgrastim (*n* = 10)				*p* value	
	WBC/ANC (any grade toxicity)	2		0				*p* = 1.0	
Hecht [23]		Placebo (*n* = 118)		Pegfilgrastim (*n* = 123)		OR (95% CI)		*p* value	
	All combined, % (95% CI)	43 (34.3–52.1)		13 (7.2–18.9)		0.19 (0.10–0.37)		*p* < 0.001	
	FOLFOX-4, % (95% CI)	*n* = 58		*n* = 61		0.11 (0.04–0.36)		*p* < 0.001	
		37.9 (25.5–51.6)		6.6 (1.8–15.9)					
	FOLFIRI, % (95% CI)	*n* = 30 50.0 (31.3–68.7)		*n* = 32 15.6 (5.3–32.8)		0.19 (0.06–0.61)		*p* = 0.0061	
	FOIL, % (95% CI)	*n* = 30 46.7 (28.3–65.7)		*n* = 30 23.3 (9.9–42.3)		0.35 (0.11–1.05)		*p* < 0.1033	
Kourlaba [25]	Severe neutropenia, % (95% CI)	Filgrastim		Pegfilgrastim				*p* value	
		32.3 (28.4–36.5)		10.4 (7.9–13.3)				*p* < 0.001	
Kurbacher [26]	Grade 3/4 neutropenia, %	Pegfilgrastim (*n* = 27)		Lipegfilgrastim (*n* = 26)					
		5.6		3.5					
Lane [27]	Duration of grade 4 neutropenia	Pegfilgrastim		G-CSF		*p* value			
	All cycles, days (95% CI)	4 (0–11)		4 (0–10)		0.55			
	A cycles, days (95% CI)	2 (0–7)		2 (0–6)		0.65			
	B cycles, days (95% CI)	6 (2–12)		6 (0–10)		0.70			
Pinter [30]	Neutropenia grade	Pegfilgrastim, *n* (%)		Placebo, *n* (%)		Difference	OR (95% CI)	*p* value	
	Grade 3/4	15 (3.6)		72 (17)		−13.5% (−18.3, −8.7)	0.18 (0.1–0.32)	< 0.001	
	Grade 4	10 (2.4)		35 (8.3)		−5.9% (−9.6, −2.2)	0.27 (0.13–0.56)	< 0.001	
	Grade 3/4 in FOLFOX-treated patients	4 (1.9)		37 (17.9)		n/a	0.09 (0.03–0.26)	n/a	
	Grade 3/4 in FOLFIRI-treated patients	11 (5.1)		35 (16.2)		n/a	0.28 (0.14–0.57)	n/a	
	Grade 3/4 in low-dose patients	4 (2.5)		28 (16.1)		n/a	0.13 (0.05–0.39)	n/a	
	Grade 3/4 in high-dose patients	11 (4.2)		44 (17.7)		n/a	0.20 (0.10–0.41)	n/a	
Skarlos [31]		Filgrastim		Pegfilgrastim		*p* value			
	Severe neutropenia, *n* (%)	34 (32)		41 (38)		*p* = 0.36			

*ANC* absolute neutrophil count, *CHOP* cyclophosphamide, doxorubicin, vincristine, and prednisone, *CI* confidence interval, *CSF* colony-stimulating factor, *FOIL* 5-fluorouracil, leucovorin, oxaliplatin, and irinotecan, *FOLFIRI* 5-fluorouracil, leucovorin, and irinotecan, *FOLFOX* 5-fluorouracil, leucovorin, and oxaliplatin, *G-CSF* granulocyte colony-stimulating factor, *n/a* not applicable, *OR* odds ratio, *Q2W* biweekly, *Q3W* every 3 weeks, *R-CHOP* rituximab, cyclophosphamide, doxorubicin, vincristine, and prednisone, *WBC* white blood cell

### Studies reporting all-cause hospitalization

Overall, six studies (two RCTs and four non-randomized studies) reported hospitalization data [20, 21, 23, 24, 28, 29] and none of them reported any statistically significant increases in all-cause hospitalization following pegfilgrastim use in Q2W setting (Table 4). Two RCTs reported hospitalization data: one RCT reported the rate of hospitalization specified due to a neutropenic event was lower in patients treated with pegfilgrastim than in those receiving placebo [23]. The second RCT reported the rate of hospitalization for any reason was higher in patients treated with pegfilgrastim than in those treated with filgrastim [20]. Of the four non-randomized studies, one study reported a higher incidence of hospitalization with pegfilgrastim compared with filgrastim [24], one study reported a lower or comparable incidence between the pegfilgrastim and the filgrastim or no prophylactic G-CSF groups (0% vs. 0% vs. 0.3% for pegfilgrastim, filgrastim, and no prophylactic G-CSF, respectively) [21], and two studies reported no significant differences between a Q3W regimen and prophylactic pegfilgrastim versus a Q2W regimen and prophylactic pegfilgrastim [28, 29].

**Table 4:** All-cause hospitalization

Study	All-cause hospitalization					
Bozzoli [20]		Total (*n* = 51)	Pegfilgrastim (*n* = 27)		Filgrastim (*n* = 24)	*p* value
	Unplanned hospitalizations per patient, *n* (%)	8/51 (16)	5/27 (19)		3/24 (13)	0.7
	Unplanned hospitalizations per cycle, *n* (%)	12/201 (6)	7/105 (7)		5/96 (5)	0.8
Donkor [21]		Pegfilgrastim < 14 days group (*n* = 126)	Pegfilgrastim > 14 days group (*n* = 25)		Filgrastim group (*n* = 90)	No CSF group (*n* = 295)
	Number of cycles with hospitalizations for FN,^a^ *n* (%)	0 (0)	0 (0)		0 (0)	1 (0.3)
Hecht [23]		Placebo (*n* = 118)	Pegfilgrastim (*n* = 123)			*p* value
	Hospitalized due to a neutropenic event, %	8	6			0.55
Hendler [24]^a,b^		Total	Group A	Group B	Group C	Group D
	Total treated, *n* (%)	231 (100)	84 (36.3)	26 (11.3)	64 (27.7)	57 (24.7)
	Hospitalized due to FN, *n* (%)	13 (5.6)	3 (3.6)	3 (11.5)	1 (1.5)	6 (10.5)
Lugtenburg [28]		R-CHOP-14			R-CHOP-21	
		< 65 years (*n* = 241)	≥ 65 years (*n* = 168)		< 65 years (*n* = 343)	≥ 65 years (*n* = 361)
	Hospitalization per group, %	26	40		22	36
	PP per intervention, %	54	58		17	32
	Hospitalization of pegfilgrastim-treated patients, %	14	23		4	12
Ng [29]		CHOP-21 (*n* = 72)			CHOP-14 (*n* = 60)	*p* value
	Hospitalization^b^, %	23.6			11.7	*p* = 0.1

^a^Group A: G-CSF 300 μg consecutive administrations during days 3–10; group B: G-CSF 300 μg consecutive administrations during days 3–7; group C: G-CSF administrations every other day for days 5, 7, 9, and 11; and group D: one administration of pegfilgrastim 6 mg on day 2

^b^Each chemotherapy cycle (not number of patients) was the unit of measurement

*CHOP* cyclophosphamide, doxorubicin, vincristine, and prednisone, *CSF* colony-stimulating factor, *FN* febrile neutropenia, *G-CSF* granulocyte colony-stimulating factor, *PP* prophylactic pegfilgrastim, *R-CHOP* rituximab, cyclophosphamide, doxorubicin, vincristine, and prednisone

### Studies reporting dose delays

Nine studies (two RCTs and seven non-randomized studies) reported on the incidence of dose delays (Table 5) [19, 23–25, 27–31]. In one RCT, the incidence of dose delays for any reason was significantly higher in patients treated with placebo than in those treated with pegfilgrastim [23], while in the second RCT, the incidence of dose delays was lower in patients treated with placebo than in those treated with pegfilgrastim, statistical significance was not assessed [30].

**Table 5:** Incidence of dose delays

Study	Dose delays								
Balducci [19]		Combined CHOP and R-CHOP Q2W				Combined CHOP and R-CHOP Q3W			
		< 65 years (*n* = 32)	65–75 years (*n* = 30)	> 75 years (*n* = 0)	Overall (*n* = 62)	< 65 years (*n* = 27)	65–75 years (*n* = 78)	75 years (*n* = 32)	Overall (*n* = 137)
	Dose delay	18.8	30.0	–	24.2	25.9	26.9	28.1	27.0
	%, 95 CI	7.2–36.4	14.7–49.4	–	14.2–36.7	11.1–46.3	17.5–38.2	13.7–46.7	19.8–35.3
Hecht [23]		Placebo (*n* = 118)			Pegfilgrastim (*n* = 123)			*p* value	
	Dose delays, % (95% CI), any reason	36.5 (27.9–45.1)			19.6 (12.7–26.5)			*p =* 0.003	
	Dose delay, % (95% CI), because of neutropenia	19.5 (12.4–26.6)			4.1 (0.6–7.6)			*p* < 0.001	
Hendler^a^ [24]		Overall patients			Group A	Group B		Group C	Group D
	Total treated, *n* (%)	231 (100)			84 (36.3)	26 (11.3)		64 (27.7)	57 (24.7)
	Treatment delays^b^	35 (3.8)			17 (5.0)	1 (0.9)		10 (3.9)	7 (3.0)
Kourlaba [25]		Filgrastim (95% CI)			Pegfilgrastim (95% CI)			*p* value	
	Treatment delays (> 2 days), % (95% CI)	42.0 (37.7–46.3)			27.6 (23.8–31.6)			*p* < 0.001	
Lane [27]		Pegfilgrastim			G-CSF			*p* value	
	Delay in next cycle, %	44.4			46.5			*p* = 0.75	
Lugtenburg [28]		R-CHOP-14			R-CHOP-21				
		< 65 years (*n* = 241)		≥ 65 years (*n* = 168)	< 65 years (*n* = 343)	≥ 65 years (*n* = 361)			
	Dose delays (per regimen), %	44		61	37	48			
	PP per intervention, %	54		58	17	32			
	Patients with a dose delay treated with pegfilgrastim, %	23.8		35	6.3	15			
Ng [29]		CHOP-14 (*n* = 60)			CHOP-21 (*n* = 72)			*p* value	
	Dose delay complications,^c^ %	16.7			19.4			*p* = 0.8	
Pinter [30]	Dose delays	Pegfilgrastim			Placebo				
	FOLFOX-treated patients, % (*n*) [95% CI]	21.3 (44) [15.9–27.5]			16.9 (35) [12.1–22.7]				
	FOLFIRI-treated patients, % (*n*) [95% CI]	27.8 (60) [21.9–34.3]			22.8 (49) [17.4–29.0]				
	Low-dose patients, % (*n*) [95% CI]	27.0 (47) [20.6–34.3]			16.9 (27) [11.4–23.6]				
	High-dose patients, % (*n*) [95% CI]	22.9 (57) [17.8–28.6]			21.8 (57) [16.9–27.2]				
Skarlos [31]		Filgrastim (*n* = 107)			Pegfilgrastim (*n* = 107)			*p* value	
	Treatment delays (> 2 days), *n* (%)	65 (61%)			61 (57%)			*p* = 0.65	

^a^Group A: G-CSF 300 μg 8 consecutive administrations during days 3–10; group B: G-CSF 300 μg consecutive administrations between days 3–7; group C: G-CSF administrations every other day for days 5, 7, 9 and 11; and group D: one administration of pegfilgrastim 6 mg on day 2

^b^The treatment delays in all the groups were due to febrile neutropenia events and nonhematological toxicity

^c^Measured per cycle

*CHOP* cyclophosphamide, doxorubicin, vincristine, and prednisone, *CI* confidence interval, *FOLFIRI* 5-fluorouracil, leucovorin, and irinotecan, *FOLFOX* 5-fluorouracil, leucovorin, and oxaliplatin, *G-CSF* granulocyte colony-stimulating factor, *PP* prophylactic pegfilgrastim, *Q2W* biweekly, *Q3W* every 3 weeks, *R-CHOP* rituximab, cyclophosphamide, doxorubicin, vincristine, and prednisone

In the seven retrospective studies, four studies evaluated filgrastim versus pegfilgrastim [24, 25, 27, 31], and three studies evaluated Q2W regimens plus prophylactic pegfilgrastim versus Q3W regimens plus prophylactic pegfilgrastim [19, 28, 29]. In three out of four studies that evaluated filgrastim and pegfilgrastim, the incidence of dose delays was lower or comparable between patients treated with pegfilgrastim and those treated with filgrastim [24, 25, 27, 31]; in one study (Kourlaba et al.) the incidence of dose delays was significantly lower between patients treated with pegfilgrastim and those treated with filgrastim (*p* <  0.001) [25] and in two of these studies (Lane et al. and Skarlos et al.), these differences were not statistically significant (*p* = 0.75 and *p* = 0.65, respectively) [27, 31] Dose delays were similar in two of the three studies that compared patients treated with prophylactic pegfilgrastim who were receiving Q2W regimens versus Q3W regimens [19, 29].

Two studies (one RCT and one retrospective study) reported dose delays as a result of neutropenia [23, 24]. In the RCT, Hecht et al. found that the incidence of dose delays due to neutropenia was significantly higher in patients receiving placebo than in those treated with pegfilgrastim (*p* <  0.001) [23]. In the retrospective study by Hendler et al., no statistically significant difference between filgrastim and pegfilgrastim was observed [24].

### Studies reporting dose reductions

Eight studies (three RCTs and five retrospective studies) reported on the incidence of dose reductions (Table 6) [19, 20, 23, 25, 28–31]. Of the three RCTs, one study evaluated filgrastim versus pegfilgrastim [20] and two studies evaluated placebo versus pegfilgrastim [23, 30]. In the study that evaluated filgrastim versus pegfilgrastim, the incidence of dose reductions was numerically lower with pegfilgrastim than with filgrastim; statistical significance was not assessed [20]. In the two studies that evaluated placebo versus pegfilgrastim, the incidence of dose reduction for any reason was numerically higher in patients receiving pegfilgrastim compared with those receiving placebo; these results were not statistically significant [23, 30]. Only one study reported dose reduction as a result of neutropenia. In the RCT, Hecht et al. found that the incidence of dose reduction because of neutropenia was significantly lower in patients treated with pegfilgrastim than in those treated with placebo (*p* <  0.02) [23].

**Table 6:** Incidence of dose reductions

Study	Dose reduction								
Balducci [19]		Combined CHOP and R-CHOP Q2W				Combined CHOP and R-CHOP Q3W			
		< 65 years (*n* = 32)	65–75 years (*n* = 30)	> 75 years (*n* = 0)	Overall (*n* = 62)	< 65 years (*n* = 27)	65–75 years (*n* = 78)	> 75 years (*n* = 32)	Overall (*n* = 137)
	Dose reductions, % (95% CI)	15.6 (5.3–32.8)	26.7 (12.3–45.9)	–	21.0 (11.7–33.2)	7.4 (0.9–24.3)	20.5 (12.2–31.2)	12.5 (3.5–29.0)	16.1 (10.3–23.3)
Bozzoli [20]		Total (*n* = 51)		Pegfilgrastim (*n* = 24)			Filgrastim (*n* = 24)		
	Reduction in dose intensity, *n* (%)	12 (23.5)		5 (20.8)			7 (26.9)		
Hecht [23]		Placebo (*n* = 118)		Pegfilgrastim (*n* = 123)			*p* value		
	Dose reduction (any reason), % (95% CI)	22 (14.8–29.2)		23.6 (16.1–31.1)			*p* = 0.77		
	Dose reduction (neutropenia), % (95% CI)	11 (5.5–16.6)		3 (0.1–6.4)			*p* < 0.02		
Kourlaba [25]		Filgrastim 95% CI		Pegfilgrastim 95% CI			*p* value		
	Dose reduction, % (95% CI)	18.5 (15.3–22.1)		10.8 (8.3–13.7)			*p* < 0.001		
Lugtenburg [28]		R-CHOP-14		R-CHOP-21					
		< 65 years	≥ 65 years	< 65 years	≥ 65 years				
	Dose reduction (per regimen), %	6	24	14	26				
	PP per intervention, %	54	58	17	32				
	Patients with a dose delay treated with pegfilgrastim, %	3	13	2.4	8.3				
Ng [29]	Dose reduction,^a^ %	CHOP-14	CHOP-21		*p* value				
		10.0	41.7		*p* < 0.0001				
Pinter [30]	Dose reductions		Pegfilgrastim		Placebo				
	FOLFOX-treated patients, % (*n*) [95% CI]		6.3 (13) [3.4–10.5]		7.7 (16) [4.5–12.2]				
	FOLFIRI-treated patients, % (*n*) [95% CI]		13.9 (30) [9.6–19.2]		9.8 (21) [6.1–14.5]				
	Low-dose patients, % (*n*) [95% CI]		7.5 (13) [4.0–12.4]		6.9 (11) [3.5–12.0]				
	High-dose patients, % (*n*) [95% CI]		12.0 (30) [8.3–16.8]		9.9 (26) [6.6–14.2]				
Skarlos [31]			Filgrastim (*n* = 107)		Pegfilgrastim (*n* = 107)		*p* value		
	Dose reductions, *n* (%)		25 (23)		25 (23)		*p* = 1		

^a^Measured per cycle

*CHOP* cyclophosphamide, doxorubicin, vincristine, and prednisone, *CI* confidence interval, *FOLFIRI* 5-fluorouracil, leucovorin, and irinotecan, *FOLFOX* 5-fluorouracil, leucovorin, and oxaliplatin, *PP* prophylactic pegfilgrastim, *Q2W* biweekly, *Q3W* every 3 weeks, *R-CHOP* rituximab, cyclophosphamide, doxorubicin, vincristine, and prednisone

Of the five retrospective studies, two studies evaluated filgrastim versus pegfilgrastim [25, 31] and three studies evaluated patients receiving a Q3W regimen and prophylactic pegfilgrastim versus a Q2W regimen and prophylactic pegfilgrastim [28, 29]. In one of the two studies that evaluated filgrastim versus pegfilgrastim, the incidence of dose reduction was significantly lower in patients treated with pegfilgrastim compared with those receiving filgrastim [25], and in the second study, the incidences of dose reduction were similar in both treatment groups [31]. In two studies, the incidence of dose reduction was numerically higher in patients receiving Q2W regimens than in those receiving Q3W regimens; statistical significance was not assessed in these studies [19, 28].

### Safety and mortality

In total, six studies (three RCTs and three retrospective studies) reported AE and serious AE data [19, 20, 22, 23, 26, 30]. Overall, only small differences in the rates of all grade AEs and serious AEs between pegfilgrastim, placebo, filgrastim, or lipegfilgrastim were observed. In the RCT, Bozzoli et al., the proportion of patients with one AE or more was numerically lower in the pegfilgrastim group compared with the filgrastim group (30% vs. 45%, respectively, (*p* < 0.3)). In the majority of the AEs reported by Bozzoli et al., the incidence of AEs was comparable in the pegfilgrastim group versus the filgrastim group (Supplementary Table 6 [Additional file 1]) [20]. In the retrospective study, Dragnev et al., the incidence of AEs reported in the pegfilgrastim group was numerically lower than in the filgrastim group (*p* < 0.6, *p* < 0.7 and *p* < 0.7 for bone pain, fever and sepsis, respectively); two patients in each group reported the incidence of bone pain. Seven patients in the filgrastim group reported the incidence of fever and sepsis, compared with two patients in the pegfilgrastim group. In Hecht et al., the incidence of grade 3 or 4 neutropenia was numerically higher in patients treated with placebo than those receiving pegfilgrastim (21% vs. 11%, respectively; statistical significance was not assessed) [23].

Only one of three retrospective studies comparing Q3W regimens and Q2W regimens had available safety data. In this study (Balducci et al.), the proportion of AEs reported was numerically higher, but not statistically significant, in patients receiving prophylactic pegfilgrastim for a Q3W regimen than in those receiving pegfilgrastim for a Q2W regimen [19]. Only two of three RCTs reported mortality data (Hecht et al. and Pinter et al.), in which comparable results were reported between patients with colorectal cancer treated with pegfilgrastim and those receiving placebo (Supplemental Table S7 [Additional file 1]) [23, 30].

### Risk of bias

The risk of bias for RCTs, measured using the Cochrane Collaboration’s tool, was low or moderate for all bias domains with the exception of performance bias in one open-label study (Supplemental Fig. S1 [Additional file 1]) [20]. In Bozzoli et al., there were no measures to blind trial participants or researchers from the knowledge of which intervention was received; therefore, the performance bias was deemed high risk [20]. The risk of bias for observational studies, measured using the Cochrane ROBINS-I tool, was low or moderate for all bias domains (Supplemental Fig. S2 [Additional file 1]).

## Discussion

This systematic review summarizes the existing literature on the efficacy, effectiveness, and safety of pegfilgrastim use among patients receiving Q2W regimens compared with those not receiving pegfilgrastim, receiving other G-CSF or receiving pegfilgrastim in a Q3W regimen. Among the 13 eligible studies evaluating heterogenous tumor types and chemotherapy regimens, most studies showed that administration of prophylactic pegfilgrastim reduced the incidence of FN in patients receiving Q2W regimens. Two out of three studies showed a lower or comparable incidence of FN with a Q2W regimen and prophylactic pegfilgrastim compared with a Q3W regimen and prophylactic pegfilgrastim; these three studies were not powered to assess statistical significance [19, 29]. In Kurbacher et al., a higher incidence of FN was observed in patients treated with pegfilgrastim compared with those receiving lipegfilgrastim [26]. However, this was an unadjusted incidence that was not statistically significant, the study population included a mixture of tumor types, and dose intensity was not accounted for. In most studies included, administration of prophylactic pegfilgrastim resulted in a decreased incidence of FN in patients across a wide variety of tumor types receiving Q2W chemotherapy regimens. In the majority of studies, the incidence of FN was low, making it difficult for statistically significant differences to be observed. Furthermore, six of the retrospective studies were not powered to conduct comparative effectiveness nor efficacy analyses, rather, they were descriptive in nature, comparing the incidence rates of FN. Although the findings indicated that administration of prophylactic pegfilgrastim reduced the incidence of FN, the results may not be statistically significant, nevertheless a decrease or comparability in the incidence of FN was observed.

Neutropenia is a relatively common disorder associated with chemotherapy [32]. Neutropenia is more frequently reported than FN, and thus assessing the rates of neutropenia enabled differences between treatment groups to be recorded. A lower incidence in grade 1–4 neutropenia was observed in the pegfilgrastim group compared with placebo or filgrastim across most studies. Six of these studies provided statistical comparisons for pegfilgrastim versus filgrastim or pegfilgrastim versus placebo. In three of these studies, there was a statistically significant decrease in the incidence of neutropenia with pegfilgrastim compared with filgrastim or placebo. In the three remaining studies, a lower incidence of neutropenia was observed with pegfilgrastim when compared with filgrastim, but these differences were not statistically significant. Similarly, a comparable incidence of neutropenia was observed with pegfilgrastim when compared with filgrastim, but these differences were also not statistically significant. In one post-hoc non-randomized subgroup analysis, the incidence of neutropenia was higher in the pegfilgrastim group compared with the filgrastim group, but the differences were not statistically significant.

The timing and methodology of neutropenia assessment were provided for five studies, and varied across studies. Further evaluations are needed to understand the impact of timing and assessment methodology of neutropenia in each study.

FN generally requires hospitalization, often resulting in a reduction in chemotherapy dose intensity due to dose delays or dose reductions [33]. Only six studies reported hospitalization data, and in these studies the incidence of hospitalization was generally low, making it difficult to identify differences between treatment groups. In all studies reporting hospitalization data, no significant differences were reported. In the five studies reporting dose delays or dose reductions, two of these studies reported a statistically significant lower incidence of dose delays and dose reductions in patients receiving pegfilgrastim compared with filgrastim. In one study, the incidence of dose reductions was lower in patients receiving Q2W and prophylactic pegfilgrastim compared with patients receiving Q3W and pegfilgrastim and this difference was statistically significant.

In this systematic literature review, all six studies that evaluated safety and the two studies that evaluated mortality, a comparable safety and mortality profile between pegfilgrastim, placebo, filgrastim and lipegfilgrastim was observed. This is consistent with previous studies that have demonstrated that pegfilgrastim had a similar safety profile and was as effective as daily filgrastim in reducing the frequency and duration of severe neutropenia [34]. The most commonly reported pegfilgrastim-related AEs across the studies included bone pain, nausea, and fever, reflecting the known safety profile of pegfilgrastim [15].

The NCCN guidelines recommend that there should be at least 12 days between a dose of pegfilgrastim and the next cycle of chemotherapy [5]. The EORTC guidelines state that pegfilgrastim can be administered with chemotherapy in patients receiving treatment at 14-day intervals [6]. The recommendation is based on phase 2 studies that reported the efficacy and safety profiles of pegfilgrastim in reducing FN among patients receiving a Q2W regimen for breast cancer [35, 36], colorectal cancer [23], lung cancer [37], or non-Hodgkin’s lymphoma [38]. These data provide additional information to support the current NCCN and EORTC guidelines on the use of prophylactic G-CSF to prevent FN in patients receiving high- or intermediate-risk chemotherapy Q2W. Furthermore, this review provides additional information to enable oncologists and payers to make evidence-based decisions.

Patients receiving prophylactic G-CSF support are likely to be different than those not receiving prophylactic G-CSF, as the decision to provide prophylaxis is dependent on several factors. Randomized trials remove the baseline confounding but could be subject to post-randomization confounding and selection bias [39]. Two of the three RCTs (Pinter et al. and Hecht et al.) showed a statistically significant decrease in the incidence of FN between pegfilgrastim and placebo [23, 30]. In the third RCT (Bozzoli et al.), the incidence of FN was also lower in the pegfilgrastim group compared with the filgrastim group, but no statistical significance was observed [20]. Study design appeared not to have an impact on the overall results, and the risk of bias assessment indicated that the majority of studies were of a high quality with a low or moderate risk of bias. However one study, a post-hoc non-randomized subgroup analysis from Skarlos et al., demonstrated a higher incidence of FN and neutropenia with pegfilgrastim compared with comparator [31]. Post-hoc subgroup analysis may not be a robust method of comparison and caution is warranted in the over interpretation of subgroup analyses [40]. In Skarlos et al., patients receiving pegfilgrastim on the same day as chemotherapy from two trials were matched to patients receiving filgrastim on days 2–10 in the same two trials. However, as per the FDA label and guidelines, it is recommended that pegfilgrastim is administered between 24 h through day 3 or 4 after the last dose of chemotherapy [11]. The concurrent administration of pegfilgrastim is convenient for patients but is associated with increased risk [12]. In this study, same day administration of pegfilgrastim led to inferior outcomes and therefore should not be recommended. The primary rationale for avoiding concurrent (same day as last chemotherapy dose) administration of G-CSF and myelosuppressive chemotherapy is that stimulation of bone marrow progenitors by the G-CSF increases the pool of precursor myeloid cells susceptible to destruction by the myelosuppressive agents [12]. The increased risk of neutropenia and FN with same day administration of G-CSF has been shown in multiple studies [12, 14, 41, 42].

Limitations of this review need to be considered. Most of the endpoints included in this review were not the primary endpoints of the evaluated studies, i.e. the studies were not powered to evaluate the effect of pegfilgrastim on these outcomes. In addition, patients in observational studies may not receive the appropriate recommended number of filgrastim administrations compared with RCTs and it was not possible to formally investigate the heterogeneity in reported effects in these studies. Furthermore, there was some publication bias with this study, as no access was available to unpublished data and no attempt was made to obtain unpublished results. Finally, the small sample sizes in some of the studies may prevent robust conclusions being drawn from these results.

## Conclusions

In most studies included in this systematic literature review, prophylactic pegfilgrastim use reduced the incidence of FN and neutropenia across a variety of non-myeloid malignancies in patients receiving a Q2W chemotherapy regimen. Comparable safety profiles were observed between pegfilgrastim, filgrastim, and placebo. These data provide additional information to support the current NCCN and EORTC guidelines on the use of prophylactic G-CSF to prevent FN in patients receiving high- or intermediate-risk chemotherapy Q2W. Additional RCTs are needed to advance our understanding among patients receiving Q2W regimens.

## Supplementary Information

**Additional file 1: Supplemental Table S1.** Embase literature search terms. **Supplemental Table S2.** Medline literature search terms. **Supplemental Table S3.** Cochrane literature search terms. **Supplemental Table S4.** Congresses in abstract literature search. **Supplemental Table S5.** Definitions of febrile neutropenia and neutropenia in included studies. **Supplemental Table S6.** Adverse events. **Supplemental Table S7.** Mortality summary. **Supplemental Figure S1.** Risk of bias: randomized controlled trials assessed by the Cochrane Collaboration’s tool. **Supplemental Figure S2.** Risk of bias: observational trials assessed by the Cochrane ROBINS-I tool

**Additional file 2.**

## Data Availability

The datasets used and analyzed during the current study are available from the corresponding author on reasonable request.

## Abbreviations

AE: Adverse event
CHOP: Cyclophosphamide, doxorubicin, vincristine, and prednisone
EORTC: European Organisation for Research and Treatment of Cancer
FN: Febrile neutropenia
FOIL: 5-fluorouracil, leucovorin, oxaliplatin, and irinotecan
FOLFIRI: 5-fluorouracil, leucovorin, and irinotecan
FOLFOX: 5-fluorouracil, leucovorin, and oxaliplatin
G-CSF: Granulocyte colony-stimulating factor
Hyper-CVAD: Hyper-fractionated cyclophosphamide, vincristine, doxorubicin, and dexamethasone
NCCN: National Comprehensive Cancer Network
Q2W: Every 2 weeks
PICOS: Population, intervention, comparison, and outcomes
Q3W: Every three weeks
RCT: Randomized controlled trial
R-CHOP: Rituximab, cyclophosphamide, doxorubicin, vincristine, and prednisone
