# Supplementary material for: Prophylactic pegfilgrastim to prevent febrile neutropenia among patients receiving biweekly (Q2W) chemotherapy regimens: a systematic review of efficacy, effectiveness and safety
Source: BMC Cancer. 2021 May 27;21:621. doi: 10.1186/s12885-021-08258-w (PMC8157684; doi:10.1186/s12885-021-08258-w)
Supplement: Supplementary file 1 — Additional file 1: Supplemental Table S1. Embase literature search terms. Supplemental Table S2. Medline literature search terms. Supplemental Table S3. Cochrane literature search terms. Supplemental Table S4. Congresses in abstract literature search. Supplemental Table S5. Definitions of febrile neutropenia and neutropenia in included studies. Supplemental Table S6. Adverse events. Supplemental Table S7. Mortality summary. Supplemental Figure S1. Risk of bias: randomized controlled trials assessed by the Cochrane Collaboration’s tool. Supplemental Figure S2. Risk of bias: observational trials assessed by the Cochrane ROBINS-I tool [file 12885_2021_8258_MOESM1_ESM.docx]

# Additional file 1

# Supplemental Table S1 Embase literature search terms

|  | **Embase search terms^a,b,c^** |
| --- | --- |
| 1 | Recombinant Granulocyte colony stimulating factor.mp OR exp recombinant granulocyte colony stimulating factor/ |
| 2 | (G-CSF OR GCSF or granulocyte stimulating factor).mp |
| 3 | Pegylat*.mp |
| 4 | 1 or 2 |
| 5 | 3 and 4 |
| 6 | ($pegfilgrastim or SD01 or neulasta or neulastim or imupeg).mp |
| 7 | Exp pegfilgrastim/ |
| 8 | (Peg-rmetHuG-CSF or polyethylene glycol-conjugated filgrastim).mp |
| 9 | 5 or 6 or 7 or 8 |
| 10 | (Tumor$ OR tumour$ OR cancer OR malignant neoplasm).mp |
| 11 | Cancer chemotherapy.mp. OR exp cancer chemotherapy/ |
| 12 | ABVD.mp |
| 13 | Hyper-CVAD.mp |
| 14 | (ACT or adoptive cellular therapy).mp |
| 15 | (CHOP 14 OR R-CHOP-14).mp |
| 16 | FOLFOX.mp |
| 17 | FOLFIRI.mp |
| 18 | (Leucovorin calcium and fluorouracil and irinotecan).mp |
| 19 | (((Fluorouracil and leucovorin) or calcium) and oxaliplatin).mp |
| 20 | (Cyclophosphamide and vincristine and doxorubicin and dexamethasone).mp |
| 21 | Epirubicin and cyclophosphamide and methotrexate and fluorouracil and docetaxel).mp |
| 22 | 10 or 11 or 12 or 13 or 14 or 15 or 16 or 17 or 18 or 19 or 20 or 21 or 22 |
| 23 | Exp neutropenia/ or neutropenia.mp or exp febrile neutropenia/ |
| 24 | 9 and 22 and 23 |
| 25 | Limit 24 to English language |

^a^An asterisk at the end of the search term will retrieve publications with the search term or any other search terms for which that is the root

^b^In search-term combinations OR will retrieve publications where there is at least one of the combined terms, and it does not exclude hits where more than one term is present

^c^Search terms in the list will be combined with OR or AND search operators in order to retrieve relevant publications with combinations of content meeting the search objectives

*ABVD* doxorubicin hydrochloride, bleomycin sulfate, vinblastine sulfate, and dacarbazine, *ACT* adoptive cellular therapy, *CHOP* cyclophosphamide, doxorubicin, vincristine, and prednisone, *FOLFIRI* 5-fluorouracil, leucovorin, and irinotecan, *FOLFOX* 5-fluorouracil, leucovorin, and oxaliplatin, *G‑CSF* granulocyte colony‑stimulating factor, *hyper‑CVAD* hyperfractionated cyclophosphamide, vincristine, doxorubicin, and dexamethasone

**Supplementary Table S2**: Initial Medline Search Terms, January 1, 2002 **–** June 30, 2019

|  | Medline Search Terms^a,b,c^ |
| --- | --- |
| 1 | Exp granulocyte colony stimulating factor OR recombinant granulocyte colony stimulating factor.mp |
| 2 | (G-CSF OR GCSF or granulocyte stimulating factor).mp |
| 3 | Pegylat*.mp |
| 4 | 1 or 2 |
| 5 | 3 and 4 |
| 6 | ($pegfilgrastim or SD01 or neulasta or neulastim or imupeg).mp |
| 7 | Pegfilgrastim.mp |
| 8 | (Peg-rmetHuG-CSF or polyethylene glycol-conjugated filgrastim).mp |
| 9 | 5 or 6 or 7 or 8 |
| 10 | (Tumor$ OR tumour$ OR cancer OR malignant neoplasm).mp |
| 11 | Cancer chemotherapy.mp. |
| 12 | ABVD.mp |
| 13 | Hyper-CVAD.mp |
| 14 | (ACT or adoptive cellular therapy).mp |
| 15 | (CHOP 14 OR R-CHOP-14).mp |
| 16 | FOLFOX.mp |
| 17 | FOLFIRI.mp |
| 18 | (Leucovorin calcium and fluorouracil and irinotecan).mp |
| 19 | (((Fluorouracil and leucovorin) or calcium) and oxaliplatin).mp |
| 20 | (Cyclophosphamide and vincristine and doxorubicin and dexamethasone).mp |
| 21 | Epirubicin and cyclophosphamide and methotrexate and fluorouracil and docetaxel).mp |
| 22 | 10 or 11 or 12 or 13 or 14 or 15 or 16 or 17 or 18 or 19 or 20 or 21 |
| 23 | Exp febrile neutropenia/ or neutropenia.mp or exp neutropenia/ |
| 25 | 9 and 22 and 23 |
| 26 | Limit 25 to English language |

^a^An asterisk at the end of the search term will retrieve publications with the search term or any other search terms for which that is the root

^b^In search-term combinations OR will retrieve publications where there is at least one of the combined terms, and it does not exclude hits where more than one term is present

^c^Search terms in the list will be combined with OR or AND search operators in order to retrieve relevant publications with combinations of content meeting the search objectives

*ABVD* doxorubicin hydrochloride, bleomycin sulfate, vinblastine sulfate, and dacarbazine, *ACT* adoptive cellular therapy, *CHOP* cyclophosphamide, doxorubicin, vincristine, and prednisone, *FOLFIRI* 5-fluorouracil, leucovorin, and irinotecan, *FOLFOX* 5-fluorouracil, leucovorin, and oxaliplatin, *G‑CSF* granulocyte colony‑stimulating factor, *hyper‑CVAD* hyperfractionated cyclophosphamide, vincristine, doxorubicin, and dexamethasone

**Supplementary Table S3**: Cochrane Library search string, January 1 2002 **–** June 30 2019

|  | Cochrane Search Terms |
| --- | --- |
| 1 | Recombinant Granulocyte colony stimulating factor.mp |
| 2 | (G-CSF OR GCSF or granulocyte stimulating factor).mp |
| 3 | Pegylat*.mp |
| 4 | 1 or 2 |
| 5 | 3 and 4 |
| 6 | ($pegfilgrastim or SD01 or neulasta or neulastim or imupeg).mp |
| 8 | (Peg-rmetHuG-CSF or polyethylene glycol-conjugated filgrastim).mp |
| 9 | 5 or 6 or 7 or 8 |
| 10 | (Tumor$ OR tumour$ OR cancer OR malignant neoplasm).mp |
| 11 | Cancer chemotherapy.mp. |
| 12 | ABVD.mp |
| 13 | Hyper-CVAD.mp |
| 14 | (ACT or adoptive cellular therapy).mp |
| 15 | (CHOP 14 OR R-CHOP-14).mp |
| 16 | FOLFOX.mp |
| 17 | FOLFIRI.mp |
| 18 | (Leucovorin calcium and fluorouracil and irinotecan).mp |
| 19 | (((Fluorouracil and leucovorin) or calcium) and oxaliplatin).mp |
| 20 | (Cyclophosphamide and vincristine and doxorubicin and dexamethasone).mp |
| 21 | Epirubicin and cyclophosphamide and methotrexate and fluorouracil and docetaxel).mp |
| 22 | 10 or 11 or 12 or 13 or 14 or 15 or 16 or 17 or 18 or 19 or 20 or 21 |
| 23 | neutropenia.mp |
| 24 | febrile neutropenia.mp |
| 25 | 23 or 24 |
| 26 | 9 and 22 and 25 |

^a^An asterisk at the end of the search term will retrieve publications with the search term or any other search terms for which that is the root

^b^In search-term combinations OR will retrieve publications where there is at least one of the combined terms, and it does not exclude hits where more than one term is present

^c^Search terms in the list will be combined with OR or AND search operators in order to retrieve relevant publications with combinations of content meeting the search objectives

*ABVD* doxorubicin hydrochloride, bleomycin sulfate, vinblastine sulfate, and dacarbazine, *ACT* adoptive cellular therapy, *CHOP* cyclophosphamide, doxorubicin, vincristine, and prednisone, *FOLFIRI* 5-fluorouracil, leucovorin, and irinotecan, *FOLFOX* 5-fluorouracil, leucovorin, and oxaliplatin, *G‑CSF* granulocyte colony‑stimulating factor, *hyper‑CVAD* hyperfractionated cyclophosphamide, vincristine, doxorubicin, and dexamethasone

**Supplemental Table S4** Congresses in abstract literature search

| Academy of Managed Care Pharmacy |
| --- |
| American College of Clinical Pharmacy |
| American Pharmacists Association |
| American Society of Clinical Oncology |
| American Society of Hematology |
| American Society of Hospital Pharmacists |
| European Hematology Association |
| European Society for Medical Oncology |
| The European Multidisciplinary Cancer Congress |
| Hematology/Oncology Pharmacy Association |
| International Society for Pharmacoeconomics and Outcomes Research (ISPOR) including European, North American, and Latin American conferences |
| Multinational Association of Supportive Care in Cancer |
| Oncology Nursing Society |
| San Antonio Breast Cancer Symposium |

# Supplemental Table S5 Definitions and timing of febrile neutropenia and neutropenia assessment in included studies

| **Study** | **Definition of FN** | Timing of FN assessment |
| --- | --- | --- |
| Balducci ^[19]^ | N/a | N/a |
| Bozzoli ^[20]^ | N/a | N/a |
| Donkor ^[21]^ | Neutropenia was defined as ANC < 1500 neutrophils/μL. Severity of neutropenia was defined as mild neutropenia (ANC 1000–1500 neutrophils/μL), moderate neutropenia (ANC 500–999 neutrophils/μL), and severe neutropenia (ANC ≤ 500 neutrophils/μL) | Assessed by collecting and reviewing patients' white blood cell count and ANC before chemotherapy and prior to the next chemotherapy administration |
| Dragnev ^[22]^ | Grade 4 neutropenia was defined as a grade ≥ 2 fever or with documented infection/sepsis | N/a |
| Hecht ^[23]^ | FN was defined as ANC < 1.0 × 10^9^/L  Grade 3/4 FN was defined as temperature ≥ 38.2°C and ANC < 1.0 × 10^9^/L on the same day or the day after | Assessed during the first four cycles of treatment. FN was determined from reported temperatures and from observed ANCs and was not based on adverse event reports. At the start of each chemotherapy cycle, blood was drawn and temperature was taken by the patient's healthcare provider |
| Hendler ^[24]^ | FN was defined as neutrophil count ≤ 0.5 × 10^9^/L associated with temperature > 38°C measured twice over a 1-hour period, or one measurement > 38.5°C | FN episodes were defined as neutrophil count ≤ 0.5 × 10^9^/L associated with temperature > 38°C measured twice over a 1-hour period, or one measurement > 38.5°C |
| Kourlaba ^[25]^ | FN was defined as temperature > 38.2°C and neutrophil count < 0.5 × 10^9^/L | N/a |
| Kurbacher ^[26]^ | N/a | N/a |
| Lane ^[27]^ | FN was defined as temperature > 38°C with ANC < 500/μL  Grade 4 neutropenia was defined as ANC < 500/μL documented on available counts | Measured from the first day ANC < 500/μL until ANC > 500/μL |
| Lugtenburg ^[28]^ | N/a | N/a |
| Ng ^[29]^ | FN was defined as a single temperature > 38.3°C or a temperature > 38°C for > 1 hour, in the presence of severe neutropenia (ANC < 0.5 × 10^9^/L)  Breakthrough FN was defined as FN occurring in spite of pegfilgrastim prophylaxis | N/a |
| Pinter ^[30]^ | Grade 3/4 FN was initially defined as ANC < 1.0 × 10^9^/L measured within 1 calendar day of an oral temperature ≥ 38°C  Grade 4 FN was defined as ANC < 0.5 × 10^9^/L measured within 1 calendar day of an oral temperature ≥ 38°C  Grade 3/4 neutropenia was defined as ANC < 1.0 × 10^9^/L  Grade 4 neutropenia was defined as ANC < 0.5 × 10^9^/L | Patients recorded their oral temperature twice daily throughout the treatment period and whenever they felt feverish. Complete blood counts were measured at the start of every cycle, at the end of the treatment visit, and if a patient experienced a temperature of ≥ 38°C |
| Skarlos ^[31]^ | FN was defined as body temperature > 38.2°C and neutrophil count < 0.5 × 10^9^/L | N/a |

*ANC* absolute neutrophil count, *FN* febrile neutropenia, *n/a* not available

**Supplementary Table 6** Safety summary

| **Study** | AEs | | | | | | | | | | | | | | | | | |
| --- | --- | --- | --- | --- | --- | --- | --- | --- | --- | --- | --- | --- | --- | --- | --- | --- | --- | --- |
| Balducci ^[19]^ | AEs | | Combined CHOP and R-CHOP Q2W | | | | | | | | | | | Combined CHOP and R-CHOP Q3W | | | | |
|  |  |  | < 65 years  (*n* = 32) | | | | | 65–75 years  (*n* = 30) | | | > 75 years (*n* = 0) | | Overall  (*n* = 62) | < 65 years  (*n* = 27) | 65–75 years  (*n* = 78) | > 75 years  (*n* = 32) | | Overall  (*n* = 137) |
|  | Patients with any grade AE leading to dose alteration or treatment discontinuation, *n*(%) | | 4 (12.5) | | | | | 8 (26.7) | | | – | | 12 (19.4) | 4 (14.8) | 24 (30.8) | 7 (21.9) | | 35 (25.5) |
|  | Patients with grade ≥ 3 AE leading to dose alteration or treatment discontinuation, *n*(%) | | 3 (9.4) | | | | | 6 (20.0) | | | – | | 9 (14.5) | 2 (7.4) | 10 (12.8) | 5 (15.6) | | 17 (12.4) |
|  | Hematological, *n* (%) | | 2 (6.3) | | | | | 2 (6.7) | | | – | | 4 (6.5) | 0 (0) | 7 (9.0) | 2 (6.3) | | 9 (6.6) |
|  | Neuropathy | | | | | | | | | | | | | | | | | |
|  | Any grade, *n* (%) | | 1 (3.1) | | | | | 4 (13.3) | | | – | | 5 (8.1) | 1 (3.7) | 5 (6.4) | 0 (0) | | 6 (4.4) |
|  | Grade ≥ 3, *n* (%) | | 0 (0) | | | | | 1 (3.3) | | | – | | 1 (1.6) | 0 (0) | 1 (1.3) | 0 (0) | | 1 (0.7) |
|  | Infections | | | | | | | | | | | | | | | | | |
|  | Any grade, *n* (%) | | 0 (0) | | | | | 2 (6.7) | | | – | | 2 (3.2) | 1 (3.7) | 3 (3.8) | 2 (6.3) | | 6 (4.4) |
|  | Grade ≥ 3, *n* (%) | | 0 (0) | | | | | 2 (6.7) | | | – | | 2 (3.2) | 0 (0) | 0 (0) | 1 (3.1) | | 1 (0.7) |
| Bozzoli ^[20]^ |  | | Total  (*n* = 51) | | | Pegfilgrastim  (*n* = 27) | | | Filgrastim  (*n* = 24) | | | | *p* value | | | | | |
|  | ≥ 1 AE, *n* (%) | | 19 (37) | | | 8 (30) | | | 11 (45) | | | | *p* = 0.3 | | | | | |
|  | Gastrointestinal, *n* (%) | | 7 (14) | | | 2 (7) | | | 5 (20) | | | | *p* = 0.2 | | | | | |
|  | Neuropathy, *n* (%) | | 4 (8) | | | 2 (7) | | | 2 (8) | | | | *p =* 1 | | | | | |
|  | Respiratory, *n* (%) | | 2 (4) | | | 1 (4) | | | 1 (4) | | | | *p =* 1 | | | | | |
|  | Cardiovascular, *n* (%) | | 1 (2) | | | 1 (4) | | | 0 (0) | | | | *p =* 1.0 | | | | | |
| Dragnev ^[22]^ |  | | Filgrastim (*n* = 25) | | | | Pegfilgrastim (*n* = 10) | | | | | *p* value | | | | | | |
|  | Bone pain, *n* | | 2 | | | | 2 | | | | | *p =* 0.56 | | | | | | |
|  | Fever, *n* | | 7 | | | | 2 | | | | | *p =* 0.7 | | | | | | |
|  | Sepsis, *n* | | 7 | | | | 2 | | | | | *p =* 0.7 | | | | | | |
| Hecht ^[23]^ |  | Placebo (*n* = 117) | | | | | | | | | | Pegfilgrastim (*n* = 124) | | | | | | |
|  | AEs, *n* (%) | All grades | Grade 3/4 | | SAE | | | | | | | All grades | | | Grade 3/4 | | SAE | |
|  | Nausea | 56 (47.9) | 5 (4.3) | | 7 (6.0) | | | | | | | 70 (56.5) | | | 5 (4.0) | | 2 (1.6) | |
|  | Diarrhea | 54 (46.2) | 8 (6.8) | | 3 (2.6) | | | | | | | 64 (51.6) | | | 14 (11.3) | | 7 (5.6) | |
|  | Fatigue | 44 (37.6) | 4 (3.4) | | 0 | | | | | | | 49 (39.5) | | | 3 (2.4) | | 0 | |
|  | Anemia | 32 (27.4) | 6 (5.1) | | 0 | | | | | | | 37 (29.8) | | | 3 (2.4) | | 2 (1.6) | |
|  | Vomiting | 26 (22.2) | 4 (3.4) | | 6 (5.1) | | | | | | | 37 (29.8) | | | 1 (0.8) | | 2 (1.6) | |
|  | Anorexia | 14 (12.0) | 1 (0.9) | | 1 (0.9) | | | | | | | 29 (23.4) | | | 2 (1.6) | | 0 | |
|  | Dehydration | 14 (12.0) | 4 (3.4) | | 6 (5.1) | | | | | | | 27 (21.8) | | | 10 (8.1) | | 14 (11.3) | |
|  | Abdominal pain | 18 (15.4) | 6 (5.1) | | 3 (2.6) | | | | | | | 23 (18.5) | | | 3 (2.4) | | 1 (0.8) | |
|  | Asthenia | 6 (5.1) | 1 (0.9) | | 0 | | | | | | | 23 (18.5) | | | 2 (1.6) | | 1 (0.8) | |
|  | Pyrexia | 11 (9.4) | 1 (0.9) | | 2 (1.7) | | | | | | | 21 (16.9) | | | 4 (3.2) | | 3 (2.4) | |
|  | Decreased weight | 8 (6.8) | 0 | | 0 | | | | | | | 19 (15.3) | | | 1 (0.8) | | 0 | |
|  | Hypokalemia | 3 (2.6) | 1 (0.9) | | 1 (0.9) | | | | | | | 18 (14.5) | | | 4 (3.2) | | 1 (0.8) | |
|  | Insomnia | 9 (7.7) | 0 | | 0 | | | | | | | 18 (14.5) | | | 1 (0.8) | | 0 | |
|  | Neutropenia | 57 (48.7) | 25 (21.4) | | 2 (1.7) | | | | | | | 17 (13.7) | | | 13 (10.5) | | 7 (5.6) | |
|  | Constipation | 13 (11.1) | 0 | | 0 | | | | | | | 16 (12.9) | | | 1 (0.8) | | 2 (1.6) | |
|  | Alopecia | 3 (2.6) | 1 (0.9) | | 0 | | | | | | | 15 (12.1) | | | 1 (0.8) | | 0 | |
|  | Bone pain | 1 (0.9) | 0 | | 0 | | | | | | | 13 (10.5) | | | 1 (0.8) | | 0 | |
|  | Dyspnea | 4 (3.4) | 0 | | 0 | | | | | | | 12 (9.7) | | | 2 (1.6) | | 0 | |
|  | Pain | 14 (12.0) | 0 | | 0 | | | | | | | 9 (7.3) | | | 0 | | 1 (0.8) | |
|  | FN | 11 (9.4) | 5 (4.3) | | 6 (5.1) | | | | | | | 4 (3.2) | | | 2 (1.6) | | 3 (2.4) | |
| Kurbacher ^[26]^ |  | | | Pegfilgrastim (*n* = 27) | | | | | | Lipegfilgrastim (*n* = 26) | | | | | | | | |
|  | Fever, *n* % | | | 2 (7.4) | | | | | | 1 (3.8) | | | | | | | | |
|  | Chills, *n* % | | | 2 (7.4) | | | | | | 0 (0) | | | | | | | | |
|  | Bone pain, *n* % | | | 2 (7.4) | | | | | | 4 (15.4) | | | | | | | | |
| Pinter ^[30]^ |  | | | Pegfilgrastim (*n* = 420) | | | | | | Placebo (*n* = 421) | | | | | | | | |
|  | Any AE | | | 344 (81.9) | | | | | | 355 (84.3) | | | | | | | | |
|  | Worse grade of ≥ 3 AE | | | 115 (27.4) | | | | | | 119 (28.3) | | | | | | | | |
|  | Serious | | | 68 (16.2) | | | | | | 55 (13.1) | | | | | | | | |
|  | Fatal | | | 10 (2.4) | | | | | | 11 (2.6) | | | | | | | | |
|  | Led to discontinuation of investigational product | | | 3 (0.7) | | | | | | 1 (0.2) | | | | | | | | |

*AE* adverse event; *CHOP* cyclophosphamide, doxorubicin, vincristine, and prednisone, *FN* febrile neutropenia; *Q2W* every 2 weeks, *Q3W* every 3 weeks, *R‑CHOP* rituximab, cyclophosphamide, doxorubicin, vincristine, and prednisone, *SAE* serious adverse event

# Supplemental Table S7 Mortality summary

| **Study** | **Mortality** | |
| --- | --- | --- |
| Hecht ^[23]^, *n* (%) | Placebo (*n* = 117) | Pegfilgrastim (*n* = 124) |
|  | 7 (6) | 7 (6) |
| Pinter ^[30]^, *n* (%) | Placebo (*n* = 421) | Pegfilgrastim (*n* = 420) |
|  | 11 (2.6) | 10 (2.4) |

**Supplemental Fig. S1** Risk of bias: randomized controlled trials assessed by the Cochrane Collaboration’s tool ^[17]^


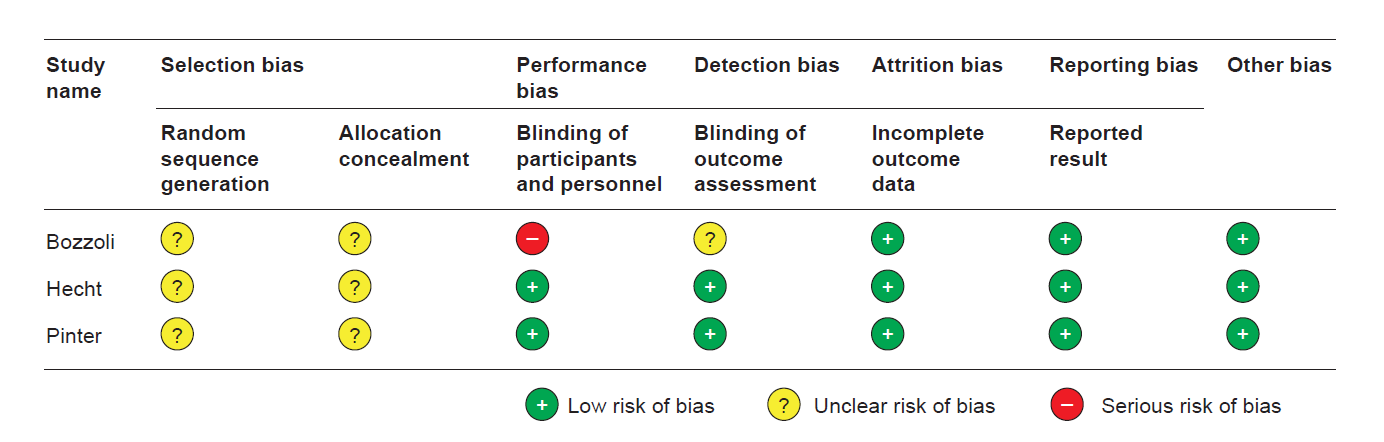


Bozzoli ^[20]^; Hecht ^[23]^; Pinter ^[30]^

**Supplemental Fig. S2** Risk of bias: observational trials assessed by the Cochrane ROBINS-I tool ^[18]^


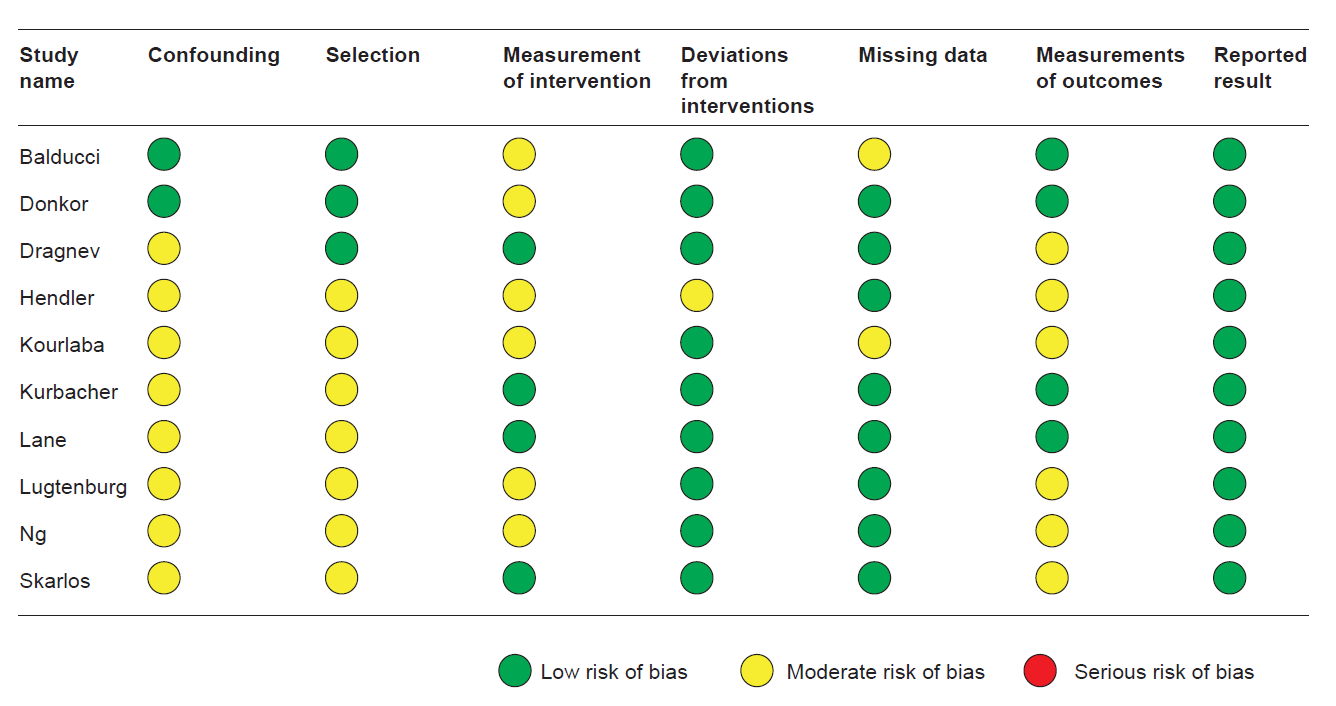


Balducci ^[19]^; Donkor ^[21]^; Dragnev ^[22]^; Hendler ^[24]^; Kourlaba ^[25]^; Kurbacher ^[26]^; Lane ^[27]^; Lugtenburg ^[28]^; Ng ^[29]^; Skarlos ^[31]^

Supplementary information

Supplementary information accompanies this paper

Additional file 1

Supplemental Table S1 Embase literature search terms

Supplemental Table S2 Medline literature search terms

Supplemental Table S3 Cochrane literature search terms

Supplemental Table S4 Congresses in abstract literature search

Supplemental Table S5 Definitions of febrile neutropenia and neutropenia in included studies

Supplemental Table S6 Adverse events

Supplemental Table S7 Mortality summary

Supplemental Fig. S1 Risk of bias: randomized controlled trials assessed by the Cochrane Collaboration’s tool

Risk of bias: observational trials assessed by the Cochrane ROBINS-I tool
