# Supplementary material for: Prophylactic pegfilgrastim to prevent febrile neutropenia among patients receiving biweekly (Q2W) chemotherapy regimens: a systematic review of efficacy, effectiveness and safety
Source: BMC Cancer. 2021 May 27;21:621. doi: 10.1186/s12885-021-08258-w (PMC8157684; doi:10.1186/s12885-021-08258-w)
Supplement: Supplementary file 2 — Additional file 2. [file 12885_2021_8258_MOESM2_ESM.docx]

**Appendix**

**Appendix A**: Initial Medline Search Terms, January 1, 2002 **–** June 30, 2019

|  | **Medline Search Terms^a,b,c^** | **Results** |
| --- | --- | --- |
| 1 | Exp granulocyte colony stimulating factor OR recombinant granulocyte colony stimulating factor.mp | 15325 |
| 2 | (G-CSF OR GCSF or granulocyte stimulating factor).mp | 23699 |
| 3 | Pegylat*.mp | 15860 |
| 4 | 1 or 2 | 23820 |
| 5 | 3 and 4 | 354 |
| 6 | ($pegfilgrastim or SD01 or neulasta or neulastim or imupeg).mp | 842 |
| 7 | Pegfilgrastim.mp | 796 |
| 8 | (Peg-rmetHuG-CSF or polyethylene glycol-conjugated filgrastim).mp | 2 |
| 9 | 5 or 6 or 7 or 8 | 1095 |
| 10 | (Tumor$ OR tumour$ OR cancer OR malignant neoplasm) | 2937354 |
| 11 | Cancer chemotherapy.mp. | 11113 |
| 12 | ABVD.mp | 1509 |
| 13 | Hyper-CVAD.mp | 242 |
| 14 | (ACT or adoptive cellular therapy).mp | 273224 |
| 15 | (CHOP 14 OR R-CHOP-14).mp | 98 |
| 16 | FOLFOX.mp | 2702 |
| 17 | FOLFIRI.mp | 1341 |
| 18 | (Leucovorin calcium and fluorouracil and irinotecan).mp | 8 |
| 19 | (((Fluorouracil and leucovorin) or calcium) and oxaliplatin).mp | 2886 |
| 20 | (Cyclophosphamide and vincristine and doxorubicin and dexamethasone).mp | 901 |
| 21 | Epirubicin and cyclophosphamide and methotrexate and fluorouracil and docetaxel).mp | 46 |
| 22 | 10 or 11 or 12 or 13 or 14 or 15 or 16 or 17 or 18 or 19 or 20 or 21 | 3176116 |
| 23 | Exp febrile neutropenia/ or neutropenia.mp or exp neutropenia/ | 40002 |
| 25 | 9 and 22 and 23 | 396 |
| 26 | Limit 25 to English language | 370 |

^a^An asterisk at the end of the search term will retrieve publications with the search term or any other search terms for which that is the root e.g. fortnight* will retrieve fortnight or fortnightly

^b^In search-term combinations OR will retrieve publications where there is at least one of the combined terms, and it does not exclude hits where more than one term is present.

^c^Search terms in the list will be combined with OR or AND search operators in order to retrieve relevant publications with combinations of content meeting the search objectives.

**Appendix B**: Embase search terms, January 1, 2002 **–** June 30, 2019

|  | **Embase Search Terms^a,b,c^** | **Results** |
| --- | --- | --- |
| 1 | Recombinant Granulocyte colony stimulating factor.mp OR exp recombinant granulocyte colony stimulating factor/ | 17058 |
| 2 | (G-CSF OR GCSF or granulocyte stimulating factor).mp | 60614 |
| 3 | Pegylat*.mp | 25371 |
| 4 | 1 or 2 | 62517 |
| 5 | 3 and 4 | 1131 |
| 6 | ($pegfilgrastim or SD01 or neulasta or neulastim or imupeg).mp | 2717 |
| 7 | Exp pegfilgrastim/ | 1163 |
| 8 | (Peg-rmetHuG-CSF or polyethylene glycol-conjugated filgrastim).mp | 2 |
| 9 | 5 or 6 or 7 or 8 | 3505 |
| 10 | (Tumor$ OR tumour$ OR cancer OR malignant neoplasm).mp | 4652921 |
| 11 | Cancer chemotherapy.mp. OR exp cancer chemotherapy/ | 428685 |
| 12 | ABVD.mp | 2865 |
| 13 | Hyper-CVAD.mp | 666 |
| 14 | (ACT or adoptive cellular therapy).mp | 319786 |
| 15 | (CHOP 14 OR R-CHOP-14).mp | 318 |
| 16 | FOLFOX.mp | 4479 |
| 17 | FOLFIRI.mp | 3273 |
| 18 | (Leucovorin calcium and fluorouracil and irinotecan).mp | 13 |
| 19 | (((Fluorouracil and leucovorin) or calcium) and oxaliplatin).mp | 3896 |
| 20 | (Cyclophosphamide and vincristine and doxorubicin and dexamethasone).mp | 8862 |
| 21 | Epirubicin and cyclophosphamide and methotrexate and fluorouracil and docetaxel).mp | 1830 |
| 22 | 10 or 11 or 12 or 13 or 14 or 15 or 16 or 17 or 18 or 19 or 20 or 21 or 22 | 4933440 |
| 23 | Exp neutropenia/ or neutropenia.mp or exp febrile neutropenia/ | 120933 |
| 24 | 9 and 22 and 23 | 1614 |
| 25 | Limit 24 to English language | 1538 |

^a^An asterisk at the end of the search term will retrieve publications with the search term or any other search terms for which that is the root

^b^In search-term combinations OR will retrieve publications where there is at least one of the combined terms, and it does not exclude hits where more than one term is present.

^c^Search terms in the list will be combined with OR or AND search operators in order to retrieve relevant publications with combinations of content meeting the search objectives.

**Appendix C**: Cochrane Library search string, January 1, 2002 **–** June 30, 2019

|  | **Cochrane Search Terms^a,b,c^** | **Results** |
| --- | --- | --- |
| 1 | Recombinant Granulocyte colony stimulating factor.mp | 1447 |
| 2 | (G-CSF OR GCSF or granulocyte stimulating factor).mp | 5744 |
| 3 | Pegylat*.mp | 2974 |
| 4 | 1 or 2 | 5744 |
| 5 | 3 and 4 | 166 |
| 6 | ($pegfilgrastim or SD01 or neulasta or neulastim or imupeg).mp | 561 |
| 8 | (Peg-rmetHuG-CSF or polyethylene glycol-conjugated filgrastim).mp | 1 |
| 9 | 5 or 6 or 7 or 8 | 665 |
| 10 | (Tumor$ OR tumour$ OR cancer OR malignant neoplasm).mp | 194546 |
| 11 | Cancer chemotherapy.mp. | 58531 |
| 12 | ABVD.mp | 587 |
| 13 | Hyper-CVAD.mp | 59 |
| 14 | (ACT or adoptive cellular therapy).mp | 12412 |
| 15 | (CHOP 14 OR R-CHOP-14).mp | 567 |
| 16 | FOLFOX.mp | 1072 |
| 17 | FOLFIRI.mp | 960 |
| 18 | (Leucovorin calcium and fluorouracil and irinotecan).mp | 55 |
| 19 | (((Fluorouracil and leucovorin) or calcium) and oxaliplatin).mp | 2358 |
| 20 | (Cyclophosphamide and vincristine and doxorubicin and dexamethasone).mp | 274 |
| 21 | Epirubicin and cyclophosphamide and methotrexate and fluorouracil and docetaxel).mp | 46 |
| 22 | 10 or 11 or 12 or 13 or 14 or 15 or 16 or 17 or 18 or 19 or 20 or 21 | 205637 |
| 23 | neutropenia.mp | 13007 |
| 24 | febrile neutropenia.mp | 4360 |
| 25 | 23 or 24 | 13007 |
| 26 | 9 and 22 and 25 | 336 |

^a^An asterisk at the end of the search term will retrieve publications with the search term or any other search terms for which that is the root

^b^In search-term combinations OR will retrieve publications where there is at least one of the combined terms, and it does not exclude hits where more than one term is present.

^c^Search terms in the list will be combined with OR or AND search operators in order to retrieve relevant publications with combinations of content meeting the search objectives.
